# Effect of vancomycin serum trough levels on outcomes in patients with nosocomial pneumonia due to *Staphylococcus aureus*: a retrospective, post hoc, subgroup analysis of the Phase 3 ATTAIN studies

**DOI:** 10.1186/1471-2334-14-183

**Published:** 2014-04-04

**Authors:** Steven L Barriere, Martin E Stryjewski, G Ralph Corey, Fredric C Genter, Ethan Rubinstein

**Affiliations:** 1 Theravance, Inc., South San Francisco, CA, 94080, USA; 2 Centro de Educación Médica e Investigaciones Clinicas (CEMIC) Norberto Quirno, Buenos Aires, Argentina; 3 Duke Clinical Research Institute and Department of Medicine, Division of Infectious Diseases, Duke University Medical Center, Durham, NC, 27710, USA; 4 Section of Infectious Diseases, Department of Internal Medicine and Medical Microbiology, University of Manitoba, Winnipeg, Manitoba, Canada

**Keywords:** Vancomycin, Trough levels, Nosocomial pneumonia, *Staphylococcus aureus*

## Abstract

**Background:**

Existing data are not consistently supportive of improved clinical outcome when vancomycin dosing regimens aimed at achieving target trough levels are used. A retrospective, post hoc, subgroup analysis of prospectively collected data from the Phase 3 ATTAIN trials of telavancin versus vancomycin for treatment of nosocomial pneumonia was conducted to further investigate the relationship between vancomycin serum trough levels and patient outcome.

**Methods:**

Study patients were enrolled in 274 study sites across 38 countries. A total of 98 patients had *Staphylococcus aureus* nosocomial pneumonia and vancomycin serum trough levels available. These patients were grouped according to their median vancomycin trough level; < 10 μg/mL, 10 μg/mL to < 15 μg/mL, and ≥ 15 μg/mL.

**Results:**

Clinical cure rates in the < 10 μg/mL, 10 μg/mL to < 15 μg/mL, and ≥ 15 μg/mL vancomycin trough level groups were 70% (21/30), 55% (18/33), and 49% (17/35), respectively (p = 0.09), and the frequencies of patient death were 10% (3/30), 15% (5/33), and 20% (7/35), respectively (p = 0.31). Renal adverse events were more frequent in the ≥ 15 μg/mL (17% [6/35]) than the < 10 μg/mL (0%) and 10 μg/mL to < 15 μg/mL (3% [1/33]) trough level groups (p < 0.01). When patients with acute renal failure or vancomycin exposure within 7 days prior to study medication were excluded, clinical cure rates in the < 10 μg/mL, 10 μg/mL to < 15 μg/mL, and ≥ 15 μg/mL vancomycin trough level groups (71% [12/17], 60% [9/15], and 27% [3/11], respectively; p = 0.04) and the number of deaths (12% [2/17], 20% [3/15], and 45% [5/11], respectively; p = 0.07) demonstrated a trend towards worse outcomes in the higher vancomycin trough level groups.

**Conclusions:**

The findings of our study suggest that higher vancomycin trough levels do not result in improved clinical response but likely increase the incidence of nephrotoxicity.

**Trial registration:**

NCT00107952 and NCT00124020

## Background

*Staphylococcus aureus* has emerged as a major cause of nosocomial pneumonia (NP), with rates of methicillin-resistant strains (MRSA) as high as 70% reported in some US studies [1]. Vancomycin, linezolid, and telavancin are currently approved for treatment of NP due to MRSA in the United States [2].

Vancomycin serum trough levels of 5–15 μg/mL have previously been targeted to optimize clinical outcome in patients with NP, although more recent literature suggests a vancomycin trough level of 15–20 μg/mL [3]. This range of trough concentrations is required to achieve the presumed optimal vancomycin AUC/MIC (area under the curve/minimum inhibitory concentration) target of > 400 for *S. aureus* with MIC values ≤ 1 μg/mL [3].

Existing data are not consistently supportive of improved clinical outcome when vancomycin dosing regimens aimed at achieving this AUC/MIC target are used [4,5]. Most recently, the results of the Phase 4 ZEPHyR trial comparing vancomycin with linezolid for MRSA NP found that higher trough levels of vancomycin were associated with lower cure rates [6].

In terms of safety, several investigations indicate that vancomycin serum levels > 15 μg/mL [5,7], and particularly > 20 μg/mL [8], can result in significant nephrotoxicity. Using Monte Carlo simulation and a pharmacokinetic/pharmacodynamic model derived from patient data, Patel et al. [9] suggested that for infections due to *S. aureus* with MIC values ≥ 1 μg/mL, vancomycin doses with an acceptable probability of AUC/MIC target attainment would result in unacceptable levels of nephrotoxicity.

Using prospectively collected data from the Assessment of Telavancin for Treatment of Hospital-Acquired Pneumonia (ATTAIN) Phase 3 clinical trials, we sought to further elucidate the relationship between measured vancomycin trough concentrations and clinical response, overall safety, incidence of nephrotoxicity, and mortality in patients with *S. aureus* NP.

## Methods

The ATTAIN trials were two identical, randomized, double-blind, comparator-controlled, parallel-group, Phase 3 studies (NCT00107952 and NCT00124020) investigating the efficacy and safety of telavancin versus vancomycin for treatment of Gram-positive NP in adult patients [10]. The institutional review board at each site approved the protocol (see Additional files 1 and 2), and all patients or their authorized representatives provided written informed consent.

Vancomycin was administered at 1 g every 12 hours for 7–21 days. Investigators were not provided with specific vancomycin trough recommendations as there were no authoritative guidelines available when the studies were conducted. Vancomycin dosing could be monitored and adjusted for weight and/or renal function according to site-specific standard procedures (while maintaining study blind).

The analysis population for the current investigation consisted of patients in the ATTAIN all-treated (AT) population (all randomized patients who received ≥ 1 dose of study medication) who had *S. aureus* NP and vancomycin serum trough levels available. These patients were grouped according to their median vancomycin trough level over the study period; < 10 μg/mL, 10 μg/mL to < 15 μg/mL, and ≥ 15 μg/mL.

Clinical cure was defined as improvement or lack of progression of baseline radiographic findings at end of study therapy (EOT) and resolution of signs and symptoms of pneumonia at the test of cure (TOC) visit. Failure was defined as persistence or progression of signs and symptoms or progression of radiological signs of pneumonia at EOT; termination of study medications due to “lack of efficacy” and initiation within two calendar days of a different potentially effective anti-staphylococcal medication; death on or after Day 3 attributable to primary infection; or relapsed infection at TOC. Renal adverse events (AEs) were defined by the investigators as acute renal failure, chronic renal failure, renal impairment, renal insufficiency, and/or by laboratory values as increased creatinine (at least 1.5× baseline to a value ≥ 1.5 mg/dL). In patients with preexisting renal dysfunction, renal adverse events were defined if they had a worsening of their baseline renal function during the study. Increased creatinine was defined programmatically using laboratory data. Kruskall-Wallis for continuous variables (e.g., age and Acute Physiology and Chronic Health Evaluation II (APACHE II) score) and Cochran-Mantel-Haenszel general association statistics for categorical variables (e.g., acute renal failure and bacteremia) were used to compare the baseline characteristics across the three vancomycin trough groups (< 10 μg/mL, 10 μg/mL to < 15 μg/mL, and ≥ 15 μg/mL). Cochran-Armitage trend test was used to evaluate clinical outcomes. No adjustment for multiple comparisons was made and a nominal two-sided p-value of < 0.05 was considered statistically significant.

## Results

Vancomycin serum trough levels were available from 98 patients with *S. aureus* NP in the ATTAIN AT population; 30, 33, and 35 patients had median vancomycin trough levels of < 10 μg/mL, 10 μg/mL to < 15 μg/mL, and ≥ 15 μg/mL, respectively. Baseline patient characteristics were generally similar between the three vancomycin trough level groups, although there was a greater proportion of patients who had failed prior antibiotic treatment in the higher vancomycin trough groups (p = 0.002; Table 1). While not statistically significantly different, more patients in the higher vancomycin trough groups had preexisting acute renal failure or multilobar pneumonia, and there were more bacteremic patients in the lower vancomycin trough groups (Table 1). Despite these differences, APACHE II scores were similar between the vancomycin trough groups (Table 1). Median trough levels were similar in patients who received ≤ 7 days or > 7 days of therapy with vancomycin (data not shown).

**Table 1:** **Baseline characteristics and clinical outcomes of ATTAIN study patients with *Staphylococcus aureus *** **nosocomial pneumonia according to vancomycin trough levels**

	**Vancomycin trough level (μg/mL), ** n (%)^*^			**p**^**†**^
	**< 10 (n = 30)**	**10 to < 15 (n = 33)**	**≥ 15 (n = 35)**	
Age (mean ± SD, year)	60 ± 20	69 ± 13	69 ± 14	0.14
APACHE II score (mean ± SD)	16 ± 5	17 ± 7	18 ± 6	0.45
Creatinine clearance ≤ 50 mL/min	10 (33)	18 (55)	14 (40)	0.22
Acute renal failure	2 (7)	5 (15)	10 (29)	0.06
Multilobar pneumonia	17 (57)	19 (58)	28 (80)	0.08
Bacteremia^‡^	6 (20)	2 (6)	2 (6)	0.10
Resident in ICU	20 (67)	24 (73)	28 (80)	0.48
Use of vasopressor/inotropic	5 (17)	5 (15)	7 (20)	0.87
Shock	2 (7)	2 (6)	4 (11)	0.68
Potentially nephrotoxic prior antibiotic therapy				
Vancomycin	11 (37)	14 (42)	17 (49)	0.63
Amikacin	1 (3)	1 (3)	2 (6)	--
Gentamicin	0	2 (6)	1 (3)	--
Prior antibiotic failure	11 (37)	18 (55)	28 (80)	0.002
MRSA	20 (67)	26 (79)	30 (86)	0.18
*Staphylococcus aureus* MIC > 1 μg/mL	0	0	1 (3)	0.41

APACHE II, Acute Physiology and Chronic Health Evaluation II; ATTAIN, Assessment of Telavancin for Treatment of Hospital-Acquired Pneumonia; ICU, intensive care unit; MIC, minimum inhibitory concentration; MRSA, methicillin-resistant *Staphylococcus aureus*; SD, standard deviation.

^*^Unless otherwise stated.

^†^Kruskall-Wallis or Cochran-Mantel-Haenszel general association.

^‡^*S. aureus* recovered from baseline blood cultures.

No statistically significant relationship was observed between clinical cure or the number of deaths and vancomycin trough level (Table 2). In addition, median length of therapy did not differ between the groups (Table 2). However, when patients with acute renal failure or vancomycin exposure within the 7 days prior to the first dose of study medication were excluded, there was a trend towards lower clinical cure rates (p = 0.04) and a greater number of deaths (p = 0.07) in the higher vancomycin trough groups (Table 2). There was a greater incidence of serious AEs and renal AEs as the vancomycin trough level increased (p = 0.03 and p < 0.01, respectively) (Table 2). This relationship was maintained when patients with acute renal failure or vancomycin exposure within the 7 days prior to the first dose of study medication were excluded (p = 0.02 and p = 0.01, respectively) (Table 2).

**Table 2:** **Clinical outcomes of ATTAIN study patients with *Staphylococcus aureus *** **nosocomial pneumonia according to vancomycin trough levels**

	**Vancomycin trough level (μg/mL), ** **n/N (%)**			p^*^
	**< 10**	**10 to < 15**	**≥ 15**	
All patients				
Clinical cure	21/30 (70)	18/33 (55)	17/35 (49)	0.09
MRSA	13/20 (65)	15/26 (58)	16/30 (53)	0.42
MRSA based on median trough levels collected up to study Day 4	14/26 (54)	11/20 (55)	8/16 (50)	0.83
Any serious adverse event	4/30 (13)	8/33 (24)	13/35 (37)	0.03
Renal adverse events^†^	0/30 (0)	1/33 (3)	6/35 (17)	<0.01
Significant increases in serum creatinine^‡^	0/30 (0)	0/33 (0)	4/35 (11)	0.02
Increase 2 to < 3 times from baseline	0/30 (0)	0/33 (0)	1/35 (3)	--
Increase ≥ 3 times from baseline	0/30 (0)	0/33 (0)	3/35 (9)	--
Deaths	3/30 (10)	5/33 (15)	7/35 (20)	0.31
Median length of therapy in days (interquartile range)	10 (6, 12)	11 (8, 14)	11 (8, 15)	0.14^¥^
Excluding patients with ARF or vancomycin exposure within 7 days^§^				
Clinical cure	12/17 (71)	9/15 (60)	3/11 (27)	0.04
Any serious adverse event	2/17 (12)	3/15 (20)	6/11 (55)	0.02
Renal adverse events^†^	0/17 (0)	0/15 (0)	3/11 (27)	0.01
Significant increases in serum creatinine^‡^	0/17 (0)	0/15 (0)	2/11 (18)	0.06
Deaths	2/17 (12)	3/15 (20)	5/11 (45)	0.07

ATTAIN, Assessment of Telavancin for Treatment of Hospital-Acquired Pneumonia; ARF, acute renal failure; MRSA, methicillin-resistant *Staphylococcus aureus*.

^*^Two-sided p-value for Cochran-Armitage test for trend; p-value based on exact test reported for sparse data (any cell < 5).

^†^Renal failure, renal insufficiency, renal impairment, creatinine increased.

^‡^≥ 1.5 mg/dL and increase to at least 1.5× baseline.

^§^Excluding patients with acute renal failure and/or vancomycin exposure within 7 days prior to the first dose of study medication.

^¥^Kruskall-Wallis.

## Discussion

This analysis of patients treated with vancomycin for NP suggests that clinical outcomes were not improved when recommended vancomycin trough levels (≥ 15 μg/mL) were achieved [3]. Importantly, all but one patient was infected with *S. aureus* isolates with MIC values ≤ 1 μg/mL, indicating that nearly all patients with a vancomycin trough level ≥ 15 μg/mL should have achieved the recommended AUC/MIC target [3]. The worse outcomes observed in the higher vancomycin trough groups may reflect differences in patient baseline characteristics between the groups. Specifically, acute renal failure was more common in the higher vancomycin trough groups, which may have influenced vancomycin distribution, and is also associated with poor clinical outcomes. Furthermore, investigators may have employed more aggressive dosing in patients with infections that were perceived to be more severe due to prior antimicrobial treatment failure or multilobar involvement, both of which were more common in the higher vancomycin trough groups.

There was more nephrotoxicity associated with the higher vancomycin troughs, consistent with previous reports [5,7,8,11] and a recent prospective study [12]. Although this may also be attributable to a greater number of patients with severe infections in the higher trough population, exclusion of the patients with major risk factors for nephrotoxicity did not change the observed trend. Concomitant medications also may contribute to the renal toxicity observed. In the study by Hidayat et al. [13], nephrotoxicity was reported in 11 of 95 subjects. Ten of these 11 subjects received concomitant nephrotoxic agents; in contrast, only 17 of the 84 subjects (20%) with no renal toxicity received nephrotoxic agents (interestingly, all 11 cases of renal toxicity occurred in the 63 subjects who had vancomycin trough levels of 15–20 μg/mL). A post hoc examination of our own data revealed prior and/or concomitant use of one or more known nephrotoxic agents in 40%, 42%, and 54% of patients in the < 10 μg/mL, 10 μg/mL to < 15 μg/mL, and ≥ 15 μg/mL vancomycin trough groups, respectively (data not shown).

The main limitation of this study is that it was a post hoc, exploratory analysis; the ATTAIN studies were not designed to evaluate the impact of vancomycin trough levels, and as such, patients were not randomized to achieve a specific trough target. In addition, the sample size may be too small to draw a definitive conclusion. However, the data were obtained from rigorously monitored, registrational, Phase 3 trials that likely capture critical information in a more systematic fashion than retrospective studies.

## Conclusion

In summary, the findings of our study suggest that higher vancomycin trough levels do not result in improved clinical response but likely increase the incidence of nephrotoxicity. Prospective controlled studies are required to further assess the validity of the current vancomycin dosing recommendations.

## Competing interests

SLB is an employee of, and holds stock/stock options for Theravance. FCG was an employee of Theravance at the time of the study. MES has received consulting fees/honoraria from Astellas, Cempra, Cerexa, Furiex, The Medicines Company, Nabriva, PRA International, Theravance, and Trius; has received other financial support (e.g., travel expenses, review activities, and manuscript preparation reimbursement) from Astellas (travel only), Cempra, and Theravance; and has received grants/grants pending from Duke University (NIH) and Theravance. GRC has served on advisory boards for Cempra, Cerexa, Inimex, Pfizer, and Trius; is a consultant for Astellas, Cempra, Cerexa, Dr Reddy’s Lab, Inimex, Merck, Pfizer, Polymedix, PRA International, Theravance, and Trius; has received grants/grants pending from Cubist, Innocoll, The Medicines Company, and Theravance; and has received other financial support (e.g., travel expenses, manuscript preparation reimbursement) from Theravance. ER has served as a consultant/on advisory boards for Astellas, Atox, Bayer, BiondVax, Merck, Ortho-McNeil, Pfizer, Theravance, and Wyeth; has provided expert testimony for Johnson & Johnson; has received grants/other research support from Astellas, Bayer-Schering, Cubist, Daiichi, Merck, Pfizer, Theravance, and Wyeth; and has received payment for lectures/speakers bureaus from Astellas, Bayer, Cubist, and Pfizer.

## Authors’ contributions

SLB was the ATTAIN study monitor, was involved in the study design and data interpretation, and was the lead author of this manuscript. FCG was involved in the statistical analysis, data interpretation, and development of the manuscript. MES was an ATTAIN study investigator, and was involved in data interpretation and development of the manuscript. GRC was an ATTAIN study co-principal investigator, and was involved in the study design, data interpretation, and development of the manuscript. ER was an ATTAIN study co-principal investigator, and was involved in the study design, data interpretation, and development of the manuscript. All authors reviewed and approved the final version of the manuscript.

## Author information

Fredric C. Genter was an employee of Theravance, Inc. at the time of the study.

## Pre-publication history

The pre-publication history for this paper can be accessed here:

http://www.biomedcentral.com/1471-2334/14/183/prepub

## Supplementary Material

Additional file 1 Institutional Review Boards/Ethics Committees by Country-Study 0015. Click here for file

Additional file 2 Institutional Review Boards/Ethics Committees by Country-Study 0019. Click here for file
