# Supplementary material for: Effect of vancomycin serum trough levels on outcomes in patients with nosocomial pneumonia due to Staphylococcus aureus: a retrospective, post hoc, subgroup analysis of the Phase 3 ATTAIN studies
Source: BMC Infect Dis. 2014 Apr 4;14:183. doi: 10.1186/1471-2334-14-183 (PMC4101862; doi:10.1186/1471-2334-14-183)
Supplement: Additional file 1 — Institutional Review Boards/Ethics Committees by Country-Study 0015. [file 1471-2334-14-183-S1.pdf]

Institutional Review Boards/Ethics Committees by Country – Study 0015

Country	Institutional Review Board/Ethics Committee
Argentina	Comite de Etica en Investigacion Clinica, "Dr. Virgilio Foglia", Tucuman 335-7° "D" (C1049AAG), Buenos Aires<br>Comite De Docencia e Investigacion Sanatorio Parque SA, Bv. Orono 860, Rosario (2000), Santa Fe<br>Departamento de Docencia e Investigacion, Comité de Revision Institucional, Sanatorio Mitre, Bartolome Mitre 2553, (C1039AAO) Cuidad de Buenos Aires<br>Instituto de Investigaciones Medicas, Dr. Alfredo Lanari, Combatientes de Malvinas 3150 (C1427ARN), Ciudad de Buenos Aires<br>Comite de Etica e Investigaciones Biomedica, Sanatorio Otamendi y Miroli S.A., Azcuenaga 870, (C1029AAP) Cuidad de Bueno Aires<br>Comite de Docencia e Investigacion, Hospital Nacional Alejandro Posadas, Marconi e Illia, (1684) Haedo, Moron, Buenes Aires<br>Comite de Etica e Investigación, Secretaria de Salud Publica de la Municipalidad de Rosario, Rueda 1100, (S2000OHV) Rosario, Sante Fe<br>Comite de Etica Medica del Hospital Frances, La Rioja 951, (C1221ACI) Cuidad de Buenos Aires<br>Comité de Docencia e Investigación del Hospital Frances, La Rioja 951 (C1221ACI) Cuidad de Buenos Aires<br>Comite de Docencia e Investigacion Hospital Escuela Eva Peron, Av. San Martin 1645, 2152 - Granadero Baigorria, Pcia Santa Fe<br>Comite de Etica en Investigación, CEMIC – Centro de Educacion Medica e Investigaciones Clinicas, Galvan 4102, (C1431FWN) Cuidad de Buenos Aires<br>Comite de Docencia e Investigacion, Clinica de Los Virreyes, Ciudad de La Paz 1157, (C1426AGU) Cuidad de Buenos Aires
Australia	Uniting HealthCare, Human Research Ethics Committee, The Wesley Hospital, Moorlands House, 451 Coronation Drive, Auchenflower QLD 4066<br>South Eastern Sydney Area Health Service, Human Research Ethics Committee – Eastern Section, Room G71, EBB, Cnr High & Avoca Streets, Randwick, NSW<br>Royal Brisbane & Women's Hospital, Health Service District, Human

Country	Institutional Review Board/Ethics Committee
	Research Ethics Committee, Room 6, G Floor Clinical Sciences Bldg., Herston Road, Herston, QLD<br><br>University of Wollongong / Illawarra Area Health Service, Human Research Ethics Committee, Northfields Ave, Wollongong, NSW<br><br>Royal Brisbane & Women's Hospital, Health Service District, Human Research Ethics Committee, Room 6, G Floor Clinical Sciences Building, Herston Road, Herston, QLD
Belgium	Comite d'Ethique, Universite Libre de Bruxelles Hopital Erasme, Route de Lennik 808, B-1070 Bruxelles<br><br>Ethisch Comite, Algemeen Stedelijk Ziekenhuis, Merestraat 80, B-9300 AALST<br><br>Comité d'ethique Medicale, CHR St. Joseph Mons, Warquignies, Avenue Baudouin de Constantinople 5, 7000 Mons<br><br>Ethisch Comité of AZ Damián, Campus Heilig Hart, Gouwelozestraat 100, 8400 Oostende<br><br>Ethisch Comité OLV Ter Linden, Graaf Jansdijik 162, 8300 Knokke-Heist<br><br>Comité d'ethique CH Notre Dame et Reine Fabiola, Grand Rue 3, 6000 Charleroi
Brazil	Irmandade da Santa Casa de Misericordia de Sao Paulo, Comite de Etica em Pesquisa em Seres Humanos, Rua Dr. Cesario Mota Junior, 112, Santa Cecilia, Sao Paulo<br><br>Comite de Etica em Pesquisa da Faculdade de Medicina de Sao Jose do Rio Preto, Av. Brigadeiro Faria Lima, 5416, Sao Jose do Rio Preto<br><br>Complexo Hospitalar Heliopolis - Comite de Etica em Pesquisa, Rua Conego Xavier, 276, Secoma – Sao Paulo - 10° andar<br><br>Universidade Federal de Santa Catarina, Comite de Etica em Pesquisa com Seres Humanos, Campus Universitario – Trindade – 88040-900, Florianópolis, Santa Catarina<br><br>Faculdade de Ciencias Medicas, Comite de Etica em Pesquisa, Caixa Postal 6111, 13083-970<br><br>Comite de Etica em Pesquisa do Hospital Geral de Vila Penteado, Dr. Jose Pangella, Av. Ministro Petronio Portela, 1642, Freguesia do O, Sao Paulo<br><br>Comite de Etica em Pesquisa do Hospital Mae de Deus, Rua Jose Alencar, 286, Porto Alegre

Country	Institutional Review Board/Ethics Committee
Canada	Humber River Regional Hospital, Research Ethics Board, 200 Church Street, Toronto, Ontario<br>Windsor Regional Hospital Research Ethics Board, 1995 Lens Avenue, Windsor, Ontario<br>Comite d'Ethique de la Recherche de L'Hopital Laval, Hopital Laval, 2725 Chemin Ste-Foy, Ste-Foy, Quebec<br>Rouge Valley Health System Research Ethics Board, 2867 Ellesmere Road, Toronto, Ontario<br>Investigational Review Board, St. John Regional Hospital 5DN, 400 University Ave St. John, New Brunswick<br>CSSS de Saint-Jerome, Comite d'ethique de la recherché de l'Hotel-Dieu de Saint-Jerome, 290 rue Montigny, St-Jerome, Quebec<br>St. Joseph's Healthcare REB, 50 Charlton Avenue East, Room H308, Hamilton, Ontario<br>Biomedical Ethics Review Board, P126-770 Bannatyne Ave, Winnipeg, Manitoba
Chile	Comité de Evaluacion Etico Cientifico – Servicio de Salud Metropolitano Sur Gran Avenida Jose Miguel Carrera 3204, Santiago<br>Comite Etico-Cientifico del Servicio de Salud Metropolitano Central, Santa Rosa 1234, Santiago de Chile<br>Comite de Etica, Direccion de Investigacion Escuela de Medicina, Pontifica Universidad Catolica de Chile, Marcoleta 391, Santiago<br>Secretaria Regional Ministerial de Salud, Comite Etico Cientifico Clinico – Octava Region, Caupolican 518 Office N° 510, Concepcion<br>Comite de Evaluacion Etico-Cientifico – Servicio de Salud Metropolitano Sur Oriente, Hospital "Dr. Sotero del Rio", Av. Concha y Toro 3459, Paradero 30 Vic. Mackenna, Puente Alto

Country	Institutional Review Board/Ethics Committee
Croatia	Local Ethics Committee of University Clinic for Infectious Diseases “Dr. Fran Mihaljevic”, Mirogojska 810000, Zagreb<br>Drug Committee of University Clinic for Infectious Diseases “Dr. Fran Mihaljevic”, Mirogojska 810000, Zagreb<br>Central Ethics Committee of Republic of Croatia, Ksaver 200a, 10000 Zagreb<br>Ministry of Health and Social Welfare of Republic of Croatia, Ksaver 200a, 10000 Zagreb<br>Drug Committee of Clinical Hospital Dubrava, Avenija Gojka Suska 6, 10000 Zagreb<br>Local Ethics Committee of Clinical Hospital Split, Spinciceva 1, 21000 Split<br>Drug Committee of Clinical Hospital Split, Spinciceva 1, 21000 Split<br>Local Ethics Committee of General Hospital “Dr. Josip Bencevic”, Andrije Stampara 42, 35000 Slavonski Brod<br>Drug Committee of General Hospital “Dr. Josip Bencevic”, Andrije Stampara 42, 35000 Slavonski Brod
Czech Republic	Multicentric Ethics Committee at the Teaching Hospital Praha Motol, V uvalu 84, 150 06 Praha 5<br>Ethics Committee at the Regional Hospital, Breclav, U nemocnice 1, 690 47 Breclav<br>Ethics Committee at the Teaching Hospital Vinohrady, Srobarova 50, 100 34 Praha 10<br>Ethics Committee at the Teaching Hospital Bulovka, Budinova 2, 180 81 Praha 8
France	CCPPRB – Paris Broussais HEGP, 96, rue Didot, 75014 Paris
Germany	Landesamt fur Gesundheit und Soziales, Geschaftsstelle der Ethikkommission des Landes Berlin, Sachsische Str. 28, D-10707 Berlin<br>Ethikkommission der Medizinischen Fakultat der Westfalishen Wilhelms-Universitat Munster und der Arztekammer Westfalen-Lippe, Von-Esmarch-Str. 62, D-48149 Munster<br>Ehtik-Kommission der Medizinische Hochschule Hannover, Carl-Neuberg-Str. 1, D-30625 Hannover

Country	Institutional Review Board/Ethics Committee
Greece	Scientific Council, General Hospital of Chest Diseases "Sotiria", 152 Mesogion Avenue, Athens<br>Scientific Council, General Hospital of Larissa 1 Tsakalof Street, Larhsa 41221<br>Scientific Council, Sismanoglio General Hospital, 1 ~ismanoglio Street, Maroussi<br>Scientific Council, "Evangelismos" General Hospital of Athens, 45-47 Ipsilantou Street, Athens<br>Scientific Council of General Hospital of Athens “George Gennimatas”, 154 Mesogeion Avenue, Athens<br>Scientific Council, AHEPA University Hospital, 1 Kiriakidi Street, Thessaloniki
India	Human Research Ethics Committee, Shri M.P. Shah Medical College, Jamnagar – 361001, Gujarat<br>Ethics Committee of KMC Hospital, N.G. Road, Attavar, Mangalore 575001<br>Institutional Review Board, Bhopal Memorial Hospital & Research Centre, Bhopal 462 038<br>Institutional Ethics Committee, Calicut Medical College, Calicut-673008, Kerala<br>Metro Ethics Review Board for Research on Human Subjects, Metro Hospital & Heart Institute, Noida, Delhi-201301<br>Poona Medical Research Foundation, 40 Sassoon Road, Pune 411001<br>Ethics Committee of M.G.M. Medical College and M.Y. Hospital, Indore-452018<br>Office of the Ethics Committee, Department of Medicine, Indira Gandhi Government Medical College, C. A. Road, Nagpur-440018, Maharashtra<br>Ethics Committee, Sardar Patel Medical College & Associate Group of Hospitals, Bikaner – 334 003, Rajasthan<br>Ethics Committee, Kamineni Hospital, L.B. Nagar, Hyderabad – 500 068<br>S.R. Kalla Memorial Ethical Committee for Human Research, 78, Dhuleshwar Garden, Behind HSBC Bank, Sardar Patel Marg, C – Scheme, Jaipur, 302001, Rajasthan<br>Monilek Hospital & Research Centre (A wing of Smt. Mohini Devi Lekhraj

Country	Institutional Review Board/Ethics Committee
	Odhrani Charitable Trust), Sector-4 Jawahar Nagar, Jaipur 302 004
Israel	Tel Aviv Sourasky Medical Center Helsinki Committee, Tel Aviv Sourasky Medical Center, Tel Aviv 64239<br>HaEmek Medical Center Helsinki Committee, HaEmek Medical Center, Afula 18101<br>Hillel Yaffe Medical Center Helsinki Committee, Hillel Yaffe Medical Center, Hadera 38100<br>Rabin Medical Center Helsinki Committee, Rabin Medical Center, Belinson, Petah Tikva 49100<br>Meir Medical Center Helsinki Committee, Meir Medical Center, Kfar Saba 44281<br>Bnai-Zion Medical Center Helsinki Committee, Bnai-Zion Medical Center, Haifa 31048<br>Soroka Helsinki Committee, Soroka University Medical Center, Beer Sheva 84101
Italy	Comitato Etico delle Aziende Sanitarie dell' Umbria, Centro Commerciale La Galleria, Piano 2, Int 37, Via Gramsci, 6, 06074 Ellera di Corciano – Perugia<br>Comitato Etico dell'Azienda Ospedaliera "Ospedali Riuniti" di Foggia, Viale Pinto, 71100 Foggia<br>Comitato Etico dell'Azienda Complesso Ospedaliero San Filippo Neri, Via Giovanni Martinotti, 20, 00135 Roma<br>Comitato Etico, Azienda Ospedaliero, Universitaria Trieste<br>Comitato Etico Locale per la Sperimentazione Clinica, dell"Ospedale Luigi Sacco, Via G.B. Grassi, 74, 20157 Milan<br>Comitato Etico Indipendente Dell'Azienda Ospedaliera "Ospedale Fatebenefratelli E Oftalmico", Corso Di Porta Nuova, 23, 20121, Milano<br>Comitato Di Bioetica Del Policlinico San Matteo, Viale Golgi, 19, 27100 Pavia<br>Comitato ETIG, Azienda Ospedaligra Di Padova, c/o Servizio Di Farmacolgia Medica, Via Girusiniani, 1, 35128 Padova<br>Comitato Etico dell'Azienda, "Ospedale Maggiore della Carita", Corso Mazzini, 18, 28100 Novara<br>Comitato Ethics I.C.P., c/o Ospedale v. Buzzi, via Castelvetro, 32, 20154

Country	Institutional Review Board/Ethics Committee
	Milan<br><br>Comitato Etico, Azienda Ospedaliera San Paolo – Polo Universitario Ufficio Segreteria – II Piano Blocco C, Via A. Di Rudini, 8, 20141, Milano
Malaysia	Research Ethics Committee (Human), Universiti Sains Malaysia, Clinical Research Platform Office, School of Dentistry, Health Campus, 16150 Kubang Kerian Kelantan<br><br>Medical Research and Ethics Committee, Faculty of Medicine, Universiti Kebangsaan Malaysia, Jalan Yacob Latif, Bandar Tun Razak, Cheras, 56000 Kuala Lumpur<br><br>Institute of Medical Ethics Committee, Institute of Medical Research (IMR), Ministry of Health, Jalan Pahang, 50588 Kuala Lumpur<br><br>Institute of Medical Research (IMR), Ministry of Health, Jalan Rumah Sakit Bangsar, 59000 Kuala Lumpur<br><br>Urusetia NIH, Institut Pengurusan Kesihatan, Jalan Rumah Sakit Bangsar, 59000 Kuala Lumpur
Malta	Health Ethics Committee, Castellania Palace, 15, Merchant’s Street, Valletta<br><br>Medicines Authority, 198, Rue D’Argens, Gzira
Peru	Comite Institucional de Etica, Universidad Peruana Cayetano Heredia, Av. Honorio Delgado 430, Lima<br><br>Comite de Etica, Clinica San Pablo, Av. El Polo 789, El Derby, Monterrico, Lima<br><br>Comite de Etica, Hospital Nacional Edgardo Rebagliati Martins, EsSalud, Av. Edgardo Rebagliati 490 – Jesus Maria, Lima<br><br>Comite Institucional de Etica, Universidad Peruana Cayetano Heredia, Av. Honorio Delgado 430, Lima
Poland	Komisja Bioetyczna przy, Centrum Medycznym Ksztalcenia Podyplomowego, ul. Mrymoncka 99/103, Warszawa<br><br>Komisja Bioetyczna Okregowej Izby Lekarskiej w Lodzi, ul. Czerwona 3, 93-005 Lodz<br><br>Komisja Bioetyczna przy Okregowej Izbie Lekarskiej, ul. Basniowa 3, Warszawska<br><br>Komisja Bioetyczna przy Okregowej Izbie Lekarskiej w Gdansku, ul. Sniadeckich 33, 80-204 Gdansk

Country	Institutional Review Board/Ethics Committee
Singapore	National Healthcare Group, NHG Domain Specific Review Board – Domain B, 6 Commonwealth Lane, Level 6 GMTI Building
Slovakia	Eticka Komisia, Fakultna NsP Trnava, A. Zarnova 11, 917 75 TRNAVA<br>Eticka Komisia, Fakultna NsP Bratislava, pracovisko Kramare, Limbova 5, 833 05 BRATISLAVA
South Africa	South African Medical Association, Research Ethics Committee, Block F Castle Walk Corporate Park, Nossob Street, Erasmuskloof Ext 3, Pretoria 0153
Taiwan	Joint Institutional Review Board, No. 201, Shih-Pai Road, Sec. 2, Taipei<br>The Institutional Review Board, Tri-Service General Hospital, No. 325, Sec. 2, Cheng-Kung Rd. Neihu, Taipei<br>Institutional Review Board, No. 110, Sec. 1, Chien-Kuo N. Road, Taichung<br>Institutional Review Board, Taichung Veterans General Hospital, No. 160, Sec. 3 Chung Kang Road, Taichung<br>The Institutional Review Board, Chi Mei Medical Center, 901 Chung Hwa Road, Yungkang City, Tainan county 710<br>Research Ethics Committee, National Taiwan University Hospital, 7 Chung-Shan South Road, Taipei<br>Joint Institutional Review Board, Taipei Veterans General Hospital, 201, Sec. 2, Shih-Pai Road, Taipei<br>Institutional Review Board of Chang Gung Memorial Hospital, 199, Tung Hwa North Road, Taipei<br>Joint Institutional Review Board, No 201, Sec. 2, Shih-Pai Road, Taipei City<br>Human Experiment & Ethics Committee, 8F, Building A, No. 100, Zihyou 1st Road<br>Sanmin District, Kaohsiung City 807<br>Human Experiment & Ethics Committee, Kaohsiung Municipal Hsiao – Kang Hospital, 482, Shan-Ming Road, Hsaio-Kang Dist 812, Kaohsiung City

Country	Institutional Review Board/Ethics Committee
Turkey	Medical, Surgical and Drug Research Ethics Committee, Hacettepe University Faculty of Medicine, Deanship Building, Sihhiye 06100, Ankara<br><br>Research Ethics Committee of Medical Faculty, Ankara University, Morphology Building, Sihhiye 06100, Ankara<br><br>Drug Research Ethics Committee, Akdeniz University Faculty of Medicine, Dumlupinar Avenue, Kampus 07070, Antalya<br><br>Ethics Committee of Istanbul University Cerrahpasa Faculty of Medicine, Deanship Building, Kocamustafapasa 34303, Istanbul<br><br>Ethics Committee of Karadeniz Technicak University Faculty of Medicine, Merkez, Trabzon<br><br>Ethics Committee of Gazi University Faculty of Medicine, Besevler 6500, Ankara
UK	Oxfordshire Regional Ethics Committee A, 2nd Floor, Astral House, Chaucer Business Park, Granville Way, Bicester<br><br>Oxfordshire Regional Ethics Committee A, Room 13, Manor House, John Radcliffe Hospital, Headley Way, Oxford<br><br>St. Thomas Hospital Research Ethics Committee, Ethics Committee Office, Block 5, South Wing, St. Thomas' Hospital, Lambeth Palace Road, London
USA	IRB for USC Health Science Campus, LAC + USC Medical Center & Health Research Association, 2020 Zonal Avenue, Room 425, Los Angeles, CA<br><br>Wright State University, Research & Sponsored Programs, 201 J University Hall, 3640 Colonel Glenn Hwy, Dayton, OH<br><br>Baystate Medical Center, Institutional Review Board, 759 Chestnut Street, Springfield, MA<br><br>University of Maryland at Baltimore Institutional Review Board, 685 W. Baltimore St., Baltimore, MD<br><br>VA Western New York Healthcare System, Institutional Review Board, 3495 Bailey Avenue, Buffalo, NY<br><br>Tulane University Health Sciences Center, Committee on the Use of Human Subjects, Tidewater Bldg., Suite 1705, 1440 Canal Street, New Orleans, LA<br><br>WIRB, 3535 Seventh Avenue SW, Olympia, WA

Country	Institutional Review Board/Ethics Committee
	Ball Memorial Hospital IRB, 2401 W. University Avenue, Muncie, IN<br>Covenant Health System, IRB, 1915 White Avenue, 7th Floor, Knoxville, TN<br>St. Francis Medial Center, 601 Hamilton Avenue, Trenton, NJ<br>Baptist Memorial Health Care Corp., Institutional Review Board, 6025 Walnut Grove Road, Suite 404, Memphis, TN<br>North Memorial Health Care IRB, 3300 Oakdale Av N, MPLS, MN<br>IRB (Subcommittee on Human Studies) of VA Long Beach Healthcare System, VA Long Beach Healthcare System, 09/151, 5901 E. Seventh Street, Long Beach, CA<br>Lehigh Valley Hospital IRB, Health Studies Research, 17th & Chew Streets, 6th Floor #38, Allentown, Pennsylvania<br>Iowa Health – Des Moines, Institutional Review Board, 1415 Woodland Avenue, Suite 218, Des Moines, IA<br>Ochsner Clinic Foundation, Institutional Review Board, 1514 Jefferson Highway, New Orleans, LA<br>Health First Institutional Review Board, 1355 S. Hickory Street, Suite 202, Melbourne, FL<br>Springfield Committee for Research Involving Human Subjects, P.O. Box 19616, Springfield, IL<br>Aurora IRB, 945 N. 12th Street, P.O. Box 342 W310, Milwaukee, WI<br>Pinnacle Health Institutional Review Board, Polyclinic Hospital, 709 Landis Building, 2501 North Third Street, Harrisburg, PA<br>University of Illinois at Chicago, Institutional Review Board, Office for the Protection of Research Subjects, 203 Administrative Office Building, M/C 672, 1737 W. Polk Street, Chicago, IL<br>Holy Spirit Hospital Institutional Review Board, 503 North 21st Street, Camp Hill, PA<br>IRB of Memorial Hospital of South Bend, 615 North Michigan Street, South Bend, IN<br>IRB of Saint Joseph's Regional Medical Center, 801 East LaSalle Avenue, South Bend, IN<br>Forsyth Medical Center, Institutional Review Board, 3333 Silas Creek Parkway, Winston-Salem, NC

Country	Institutional Review Board/Ethics Committee
	ETSU/VA Institution Review Board, East Tennessee State University, Box 70565, Johnson City, TN
	Sharp Health Care, Office for the Protection of Research Participants, 8695 Spectrum Center Boulevard, San Diego, CA
	Winchester Medical Institutional Review Board, 1840 Amherst Street, Winchester, VA
	Sutter Health Central Area, Institutional Review Committee, 2801 Capitol Avenue, Ste. 400, Sacramento, CA
	Sentara Virginia Beach General Hospital, Institutional Review Board, 1060 First Colonial Road, Virginia Beach, VA
	Human Studies Committee, Washington University School of Medicine, 660 S. Euclid Avenue, Box 8052, St. Louis, MO
	Medstar Research Institute, Institutional Review Board, 6495 New Hampshire Ave., Ste. 201, Hyattsville, MD
	Covenant Health Systems, Institutional Review Board, 1915 White Avenue, Knoxville, TN
	University of Texas, Committee for the Protection of Human Subjects, Office of Research Support Committees, University Center Tower, 7000 Fannin, Suite 750, Houston, TX
	St. Bernards Medical Center, Institutional Review Board, 225 E. Jackson, Jonesboro, AR
	Health Sciences Institutional Review Board, 125 Folk Hall, One Hospital Drive, Columbia, MO
	Baptist Health System Institutional Review Board, 137 Blount Avenue, Knoxville, TN
	Mercy Hospital Institutional Review Board, 3663 South Miami Avenue, Miami, FL
	Baptist Health System, Institutional Review Board, 730 Main, Suite 324, San Antonio, TX
	Baptist Health System Institutional Review Board, 137 Blount Avenue, Knoxville, TN
	Patient Advocacy Council, Institutional Review Board, 601 Bel Air Boulevard, Suite 315, Mobile, AL
	Institutional Review Board, Good Samaritan Hospital, c/o Medical Staff Office, 2425 Samaritan Drive, San Jose, CA

Country	Institutional Review Board/Ethics Committee
	Eastern Maine Medical Center, Human Rights Committee, 489 State Street, Bangor, Maine
