# Supplementary material for: Effect of vancomycin serum trough levels on outcomes in patients with nosocomial pneumonia due to Staphylococcus aureus: a retrospective, post hoc, subgroup analysis of the Phase 3 ATTAIN studies
Source: BMC Infect Dis. 2014 Apr 4;14:183. doi: 10.1186/1471-2334-14-183 (PMC4101862; doi:10.1186/1471-2334-14-183)
Supplement: Additional file 2 — Institutional Review Boards/Ethics Committees by Country-Study 0019. [file 1471-2334-14-183-S2.pdf]

Institutional Review Boards/Ethics Committees by Country – Study 0019

Country	Institutional Review Board/Ethics Committee
Argentina	Comite de Etica en Investigacion Clinica, “Dr. Virgilio Foglia”, Tucuman 335-7° “D“, (C1049AAG) Buenos Aires
	Comite de Docencia e Investigacion, Hospital General de Agudos, Dr. Cosme Argerich, Av. Almirante Brown 240, C1155ADP Buenos Aires
	Comite de Bioetica, Hospital General de Agudos, Dr. Cosme Argerich, Av. Almirante Brown 240, C1155ADP Buenos Aires
	Comite Hospitalario de Bioetica, Hospital F.J. Muniz, Uspallata 2272, (C1282AEN) Buenos Aires
	Comite de Docencia e Investigacion, Sanatorio San Jose, Sanchez de Bustamante 1674, (C1425DHU), Buenos Aires
	Departamento de Docencia e Investigacion, Hospital Aleman, Av. Pueyrredon 1640, (1118), Ciudad de Buenos Aires
	Comité de Bioetica Medica, Av. Pueyrredon 1640, (1118) Buenos Aires
	Comite Institucional de Etica de, Investigacion en Salud del Sanatorio Allende, Hipolito Yrigoyen 384, (5000) Cordoba
	Comite de Etica del Hospital de Clinicas, Av. Cordoba 2351, (C1120AAR) Buenos Aires
	Direccion de Docencia e Investigacion, Av. Cordoba 2351, (C1120AAR) Buenos Aires
	Hospital Privado Centro Medico de Cordoba, Departamento de Docencia e Investigacion, Comite De Revision Interna, Av. Naciones Unidas 346, Parque Velez Sarfield (5016), Cordoba
	Fundacion Medica de Mar del Plata, Departmento de Docencia e Investigacion, Cordoba 4545, (7600) Mar del Plata, Buenos Aires
	Comite de Etica del Instituto A. Roffo, Av. San Martin 5481, (C1417DTB) Buenos Aires
	Comite de Docencia e Investigacion, Instituto A. Roffo, Av. San Martin 5481, (C1417DTB) Ciudad de Buenos Aires
	Comite de Etica, Hospital General de Agudos Carlos G. Durand, Av. Diaz Velez 5044, (C1405DCR) Buenos Aires
	Comité de Dodencia e Investigacion, Hospital General de Agudos Carlos G. Durand, Av. Diaz Velez 5044, (C1405DCR) Buenos Aires

Country	Institutional Review Board/Ethics Committee
Australia	Melbourne Health Human Research Ethics Committee, Charles Connibere Building, The Royal Melbourne Hospital, Flemington Road, Parkville, Victoria<br>Eastern Health Research and Ethics Committee, Level 2, Clive Ward Centre, 16 Arnold Street, Box Hill, Victoria<br>Human Research Ethics Committee, Level 4 The Queen Elizabeth Hospital, Department of Intensive Care Medicine, 28 Woodville Road, Woodville, South Australia<br>Sydney West Area Health Service (Westmead Campus), Human Research Ethics Committee, Research Office, Clinical Sciences, Westmead Hospital Campus, Westmead, New South Wales<br>Bendigo Health Human Research Ethics Committee, PO Box 126, Bendigo, Victoria<br>Human Research Ethics Committee, Research Office, Level 4, Vindin House, Royal North Shore Hospital, Pacific Hwy, St. Leonards, New South Wales<br>ACT Health and Community Care, Human Research Ethics Committee, 11 Moore Street, Canberra, ACT<br>Southern Health Human Research Ethics Committee, 246 Clayton Road, Clayton Victoria<br>Human Research Ethics Committee, Level 6, de Lacy Bldg., Victoria Street, Darlinghurst, NSW
Brazil	Comite de Etica em Pesquisa, Instituto de Assistencia Medica ao Servidor Publico Estadual, Av. Ibirapuera, 981 - 6° andar – sl. 605, Sao Paulo<br>Comite de Etica em Pesquisa – CEP/ISCMPA, Rua Prof. Annes Dias, 285, Porto Alegre – Rio Grande do Sul<br>Comite de Etica em Pesquisa do, Hospital Professor Edmundo Vaconcelos, Rua Borges Lagoa, 1450, CEP: 04038-905, Sao Paulo<br>Comite de Etica em Pesquisa do Hospital Guilherme Alvaro, Rua Oswaldo Cruz, 197 – Boqueirao, Santos, SP – CEP: 11045-904<br>Comite de Etica em Pesquisa da Faculdade de Medicina do ABC, Av. Principe de Gales, 821, Santo Andre, SP – CEP: 09060-650<br>Comite de Ethica em Pesquisa do Instituto Dante Pazzanese de Cardiologia, Av. Dr. Dante Pazzanese, 500., Ibirapuera, Sao Paulo

Country	Institutional Review Board/Ethics Committee
Bulgaria	Local Ethics Committee, Multifunctional Hospital for Active Treatment and Emergency Medicine (MHATEM) “N.I. Pirogov”, 21, Totleben Blvd, 1606 Sophia<br>Local Ethics Committee, University Multifunctional Hospital for Active Treatment “Sv. Anna”, Clinic of Anesthesiology and Intensive Care, 1 “Dimitar Mollov” Str., 1709 Sofia<br>Local Ethics Committee, Multifunctional Hospital for Active Treatment “Prof Kirkovich” 0, “Stoletov” Blvd, 6003 Stara Zagora<br>Local Ethics Committee, University Multifunctional Hospital for Active Treatment, 11 “Armeiska” Str. 6003 Stara Zagora<br>Local Ethics Committee, Regional Hospital for Pulmonary Diseases, First Pulmonology Department, 1 “Aleia Lilia” Str., 7002 Rousse<br>Local Ethics Committee, University Multifunctional Hospital for Active Treatment (UMHAT) “Sv. Marina”, 1 “Hristo Smirnenski” Str., 9010 Varna<br>Local Ethics Committee, University Multifunctional Hospital for Active Treatment “Sv. Georgi”, 15A “Vassil Aprilov” Str., 4000 Plovdiv<br>Local Ethics Committee, Specialized Hospital for Active Treatment of Pneumo-Phtysiatric Diseases, 64 “General Gurko” Str., 8000 Bourgas
Canada	Maisonneuve Rosemont Hospital Ethic Board, 5415 boul de l'Assomption, Montreal, Quebec<br>University of Calgary, Conjoint Health Research Ethics Board, Office of Medical Bioethics, Heritage Medical Research Bldg, Room 93, 3330 Hospital Dr. N.W., Calgary, Alberta<br>Fraser Health Research Ethics Board, #300-10334 152A Street, Surrey, BC<br>University of British Columbia Clinical Research Ethics Board, Research Pavillion, Room 210-828 West 10th Avenue, Vancouver, British Columbia<br>Queen’s University Health Services & Affiliated Teaching Hospitals, Human Research Ethics Board, Office of Research Services, Fleming Hall – Room 301, Kingston, Ontario<br>Brandon Regional Health Authority Ethics Committee, Brandon Regional Health Center, 150 McTavish Avenue East, Brandon, Manitoba

Country	Institutional Review Board/Ethics Committee
Chile	Comite de Etica-Cientifico Servicio de Salud Araucania Sur, General Mackenna 51, Temuco<br>Comite de Etica-Cientifica - Servicio de Salud Metropolitano Oriente, Av Salvador 364, Providencia, Santiago<br>Comite de Etica de la Investigacion, Organismo asesor de Direccion Del Servicio de Salud Metropolitano Norte, CEI-SSMN, Maruri 272, Santiago<br>Comite Etica, Clinica Davila, Recoleta 464, Santiago
China	Ethics Committee of the First Affiliated Hospital, Sun Yat-Sen University, 58 Zhongshan Road 2, Guangzhou 510080, Guangdong Province<br>Ethics Committee of Beijing Anzhen Hospital of the capital university of medical sciences, An Zhen Li, An Ding Men Wai, Chao Yang District, Beijing<br>Ethics Committee of The Second Affiliated Hospital of Medical College, Zhejiang University, 88 Jiefang Road, Hangzhou 310009, Zhejiang Province<br>Ethics Committee of The First Affilated Hospital of People’s Liberation Army General Hospital, No. 51 Fucheng Road, Hai Dian District, Beijing<br>Ethics Committee of Nanjing General Hospital of Nanjing Military Command, 305 East Zhongshan Road, Nanjing 210002, Jiangsu Province<br>Ethics Committee of The First Affiliated Hospital of Medical College, Zhejiang University, 79 Qingchun Road, Hangzhou 310003, Zhejiang Province<br>Ethics Committee of The Third Affiliated Hospital of Sun Yat-Sen University, Gang Ding, Shi Pai, Guangzhou, 510060, Guangdong Province<br>Ethics Committee of The First Affiliated Hospital of Nanjing Medical University, 300 Guangzhou Road, Nanjing 210029, Jiangsu Province<br>Ethics Committee of The Second Hospital of Tianjin Medical University, No.23 Ping Jiang Dao, He Xi District, Tianjin, 30021 1<br>E. C. Shanghai Jiao Tong University Affiliated First People's Hospital, 85 Wujin Road, Shanghai<br>Ethics Committee of Nanjing Drum Tower Hospital, 58 Zhongshan Road 2, Guangzhou 510080, Guangdong Province<br>Ethics Committee of Nanjing Drum Tower Hospital, 321 Zhongshan Road, Nanjing 210008, Jiangsu Province

Country	Institutional Review Board/Ethics Committee
	IRB of West China Hospital, 37# Guo Xue Xiang, Chengdu, Sichuan<br>The Ethics Committee of The Third Xiang Ya Hospital, He Xitong Zi Po Road, Chang Sha City<br>Ethics Committee of Zhongshan Hospital, Fudan University, No.180 Feng Lin Road, Xu Hui District, Shanghai<br>The Ethics Committee of Tong Ji Hospital of Tong Ji Medical College of Hua Zhong University of Science & Technology, No.1095 Jie Fang Road, Wu Han City<br>Ethics Committee of Shanghai Changhai Hospital, No.174 Changhai Road, Shanghai<br>The IRB of Hainan Provincial People's Hospital, Xiuhua Road 19, Xiuying District, Haikou<br>Ethics Committee of Sir Run Run Shaw Hospital, No. 3 Qing Chun Road East, Hangzhou, Zhejiang Province<br>The First Affiliated Hospital of China Medical University, No.155 Nanjing Bei Road, Heping Area, Shenyang, Liaoning<br>IRB of The First Affiliated Hospital of Peking University, No. 8, Xi Shen Ku Road, Xi Cheng Area, Beijing<br>Ethics Committee of General Hospital of Shenyang Military Region, No. 83 Wen Hua Road, Shen He District, Shenyang<br>Ethics Committee of Xijing Hospital of The 4th Military Medical University, No. 15, Chang Le Xi Road, Xian<br>Peking Union Medical College Hospital Ethics Committee, No. 1 Shuaifuyuan Wangfujing, Dongcheng Area, Beijing
Croatia	Drug Committee of Clinical Hospital Centre Zagreb, Kispaticeva 12, 10000 Zagreb<br>Central Ethics Committee of Republic of Croatia, Ksaver 200a, 10000 Zagreb<br>Ministry of Health and Social Welfare of Republic of Croatia, Ksaver 200a, 10000 Zagreb<br>Local Ethics Committee of University Hospital for Infectious Diseases "Dr. Fran Mihaljevic", Mirogojska 8, 10000 Zagreb<br>Drug Committee of University Hospital for Infectious Diseases "Dr. Fran Mihaljevic", Mirogojska 8, 10000 Zagreb

Country	Institutional Review Board/Ethics Committee
Czech Republic	Multicentric Ethics Committee at the Teaching Hospital Praha Motol, V uvalu 84, 150 06 Praha 5<br>Ethics Committee at the Hospital Vitkovice, Zaluzanskeho 1192/15, 703 84 Ostrava-Vitkovice<br>Multicentric Ethics Committee at the Teaching Hospital Praha Motol, V uvalu 84, 150 06, Praha 5<br>Ethics Committee at the Regional Hospital Tabor, Trida Kapitana Jarose 2000, 390 03 Tabor<br>Ethics Committee at the Teaching Hospital Hradec Kralove, Sokolska 581, 500 05, Hradec Kralove 5
Estonia	Tallinn Medical Research Ethics Committee/National Institute for Health Development, Hiiu 42, Tallinn
France	C.C.P.P.R.B. – Paris-Broussais-HEGP, 96, rue Didot, 75014 Paris
Georgia	Local Ethics Committee of Ltd I. Javakhishvili Tbilisi State University Clinic, 60, D Agmashenebeli Ave, Tbilisi<br>Local Ethics Committee of Tbilisi State Medical University of Institute of Postgraduate Medical Education and Continuous Professional Development, 29, Chavchavadze Ave., Tbilisi<br>Local Ethics Committee of Legal Entity of Public Low “N. Kipshidze Central Clinical Hospital” of Tbilisi State Medical University, 29 Vaja Pshavela Ave., Tbilisi<br>Local Ethics Committee of Union Clinical City-First Hospital, 9 Tsinandali Street, Tbilisi<br>Local Ethics Committee of JSC Academic O. Gudushauri National Medical Centre, 18/20 Lubliana Street, Tbilisi
Germany	Landesamt fur Gesundheit und Soziales Geschaftsstelle der Ethikkommission des Landes Berlin, Sachsische Str. 28, D-10707 Berlin<br>Ehtik-Kommission der Medizinischen Hochschule Hannover, Carl-Neuberg-Str. 1, D-30625 Hannover<br>Arztekammer Mecklenburg-Vorpommern Ethikkommission, August-Bebel-Str. 9A, 18055 Rostock

Country	Institutional Review Board/Ethics Committee
Greece	Scientific Council, University Hospital of Patra, Red Cross General Hospital of Athens, 1 Erithrou Stavrou and Athanasaki Street, Athens<br>Administrative Board “Tzaneion” General Hospital of Piraeus, Afentouli 1 and Tzanni Street, Pireaus<br>Scientific Council, General Hospital of Chest Diseases "Sotiria", 152 Mesogion Avenue, Athens<br>“KAT: General Hospital, 2 Nikis Street, Kifissia 14561, Athens<br>Scientific Committee, 251 Airforce General Hospital, 3 P. Kanellopoulou Street, Athens<br>Scientific Council, General Hospital of Lamia, Terrna Papasiopoulou Street, Lamia
Israel	E. Wolfson Medical Center Helsinki Committee, E. Wolfson Medical Center, Holon<br>Sheba Medical Center Helsinki Committee, Sheba Medical Center, Tel Hashomer<br>Assaf Harofeh Medical Center Helsinki Committee, Assaf Harofeh Medical Center, Zerifin<br>Barzilai Medical Center Helsinki Committee, Barzilai Medical Center, Ashkelon<br>Kaplan Medical Center Helsinki Committee, Kaplan Medical Center, Rehovot<br>Western Galilee Hospital Helsinki Committee, Western Galilee Hospital – Nahariya, Nahariya<br>Rambam Medical Center Helsinki Committee, Rambam Medical Center, Haifa
Korea	IRB of Korea University Guro Hospital, 80, Guro-dong, Guro-gu, Seoul<br>IRB of Pusan National University Hospital, 10, Ami-dong 1-ga, Seo-gu, Busan<br>IRB of Korea University Anam Hospital, 126-1, Anam-dong 5-ga, Seongbuk-gu, Seoul<br>IRB of Yeungnam University Medical Center, 317-1 Daemyeong-dong, Nam-gu, Daegu<br>IRB of Ajou University Hospital, San 5, Woncheon-dong, Yeongtong-gu Suwon-si, Gyeonggi-do

Country	Institutional Review Board/Ethics Committee
	The Intitutional Review Board, Ewha Womans University Mokdong Hospital, 911-1, Mok-dong, Yangcheon-gu, Seoul<br>IRB of Kangnam St. Mary's Hospital, 505, Banpo-dong, Seocho-gu, Seoul<br>IRB of Wonju Christian Hospital, 162, Ilsan-dong, Wonju, Kangwon-do<br>The Institutional Review Board, Ulsan University Hospital, 290-3 Jeonha-dong, Dong-gu, Ulsan<br>The Institutional Review Board, Gil Medical Center, Gachon Medical School, 1198 Kuwol-dong, Namdong-gu, Incheon<br>The Institutional Review Board, Severance Hospital, Yonsei University College of Medicine, 134 Sinchon-dong, Seodaemun-gu, Seoul<br>The Institutional Review Board, KyungHee University Medical Center, 1 Hoeki-dong, Dongdaemun-gu, Seoul
Latvia	State Agency of Medicines Latvia, Riga, Jersikas 15<br>Central Ethics Committee, 11 Novembra Krastmala 29, Riga LV-1050
Lebanon	The Institutional Review Board, Faculty of Medicine, American University of Beirut, Riad El Solh, Beirut<br>Medical Ethics Committee, St. George University Hospital, Achrafieh<br>Ethics Committee, Hotel Dieu de France University Hospital , Alfred Naccache Ave, Achrafieh
Lithuania	Lithuanian Bioethics Committee, Vilniaus str. 33-230, LT-1119 Vilnius<br>State Medicine Control Agency, Traku str. 14, LT-01132 Vilnius

Country	Institutional Review Board/Ethics Committee
Mexico	Comite de Etica del Hospital Central Dr. Ignacio Morones Prieto, Avenida Venustiano Carranza No. 2395, Zona Universitaria, 78240, San Luis Potosi,<br><br>Comite de Etica del Hospital Civil “Fray Antonio Alcalde”, Calle Hospital No. 278 S.H., 44100, Guadalajara, Jalisco<br><br>Comite de Etica del Hospital General Dr. Aurelio Valdivieso, Calzada Porfirio Diaz 400 Colonia Reforma 68050, Oaxaca<br><br>Comite de Etica Hospital San Jose TEC de Monterrey, Morones Prieto #3000 Pte., Colonia Los Doctores, 64710, Monterrey, Neuvo Leon<br><br>Comite de Etica del Hospital Central Universitario de Chihuahua, Rosales 3302, Colonia Obrera, 31350, Chihuahua<br><br>Comite de Investigacion para Estudios es Humanos, Puente de Piedra # 150. Toriello Guerra, Tlalpan C.P. 14050<br><br>Comite de Ensenanza, Investigacion, Capacitacion Etica y Admision del Centenario Hospital Miguel Hidalgo, Galeana Sur 465, Col Obraje, CP 20230 Aguascalientes Ags.
Philippines	Research Implementation Development Office, UP-Philippines General Hospital, Taft Avenue, Manila<br><br>Research and Ethics Committee, Veterans Memorial Medical Center, North Avenue, Diliman, Quezon City<br><br>Ethics Review Committee, Lung Center of the Philippines, Quezon Avenue, Quezon City, Manila<br><br>Ethics Review Board, St. Luke’s Medical Center, E. Rodriguez Sr. Avenue, Quezon City<br><br>Makati Medical Center - Institutional Review Board, Amorsolo Street, Legaspi Village, Makati City<br><br>Research & Ethics Committee, Manila Doctors Hospital, 667 United Nations Avenue, Manila<br><br>Institutional Review Board, Quirino Memorial Medical Center, Katipunan Road Corner P. Tuazon Avenue, 1109 Quezon City

Country	Institutional Review Board/Ethics Committee
Poland	Komisja Bioetyczna Lubelskiej Isby Lekarskiej, ul. Chmielna 4, 20-079 Lublin<br>Komisja Bioetyczna przy Centrum Medycznym Ksztalcenia Podyplomowego, ul. Marymoncka 99/103, Warszawa<br>Uczelniana Komisja Etyki Badan Naukowych, Al. Kosciuszki 4, 80-419 Lodz<br>Komisja Etyczna Nadzoru Nad Badaniami na Ludziach Centralnego Szpitalia Klinicznego MSWiA, ul. Woloska 137, 02-507 Warszawa<br>Komisja Bioetyczna przy Okregowej Izbie Lekarskiej, ul.Krupnicza 11a, 31-123 Krakow<br>Komisja Bioetyczna przy Okregowej Izbie Lekarskiej, ul.Krupnicza 11a, 31-123 Krakow
Portugal	Comissao de Etica para a Investigacao Clinica (CEIC), Parque de Saude de Lisboa – Avenida do Brasil, 53, 1749-004 Lisboa
Romania	National Ethics Committee, 48 Aviator Sanatescu St., Sector 1, 011478 Bucharest

Country	Institutional Review Board/Ethics Committee
Russia	Ethics Committee at the Federal Body of Medicines Quality Contol, 8 Petrovsky Blvd., bld. 3, Moscow, 127051<br>Local Ethics Committee of Municipal Health Care Institution Municipal Clinial Hospital #13 1/1, Velozavodskaya str., Moscow, 115280<br>Ethics Committee at Roszdravnadzor, 4 Slavyanskaya Sq., bld. 1, 109074 Moscow<br>Local Ethics Committee of Municipal Health Care Institution of Moscow Municipal Clinical Hospital #11 of Health Department of Moscow, 6 Dvintsev Street, 127018, Moscow<br>Local Ethics Committee of the Central Clincal & Diagnostics Federal State Institution “National Medicine & Surgery Center named after N.I. Pirogov” under Federal Agency for Health Care & Social Development, 70 Nizhnyaya Pervomayskaya, 105203 Moscow<br>Local Ethics Committee of State Institution “Scientific Research Institute of Clinical & Experimental Lymphology of RAMS, Siberian Branch, 2 Timakov str., 630117 Novosibirsk<br>Local Ethics Committee of Municipal Health Care Institution “Municipal Clinical Hospital #2”, 21 Polzunov str., Novosibirsk, 630051<br>Local Ethics Committee of Federal State Institution Novosibirsk Scientific Research Institute of Blood Circulation Pathology named after academician E.N. Meshalkin, 15 Rechkunovskaya str., 630055 Novosibirsk<br>Local Ethics Committee of the Saint-Petersburg State Medical Institution “Municipal Hospital #26”, 2 Kostyushko str., Saint Petersburg 196247<br>Local Ethics Committee of Municipal Health Care Institution Clinical Hospital of Emergency Care named after N.V. Solovyev, 11 Zagorodny Sad str., 150003, Yaroslavl<br>Local Ethics Committee of the State Medical Institution of Moscow “Clinical Hospital #7”, 4 Kolomensky proezd, Moscow 115446

Country	Institutional Review Board/Ethics Committee
Serbia	Medicines and Medical Devices Agency of Serbia, Vojvode Stepe 458, 11152 Belgrade<br>Ethics Committee, Clinic for Pulmonary Diseases and TBC “Knez Selo”, ZP 18204 Gornji Metejevac, 18 000 Nis<br>Ethics Committee, Clincal Center Kragujevac, Zmaj Jovina 30, 34 000 Kragujevac<br>Medicines and Medical Devices Agency of Serbia, Vojvode Stepe 458, 11152 Belgrade<br>Ethics Committee, Clinical Center Serbia, Pasterova 2, 11000 Belgrade<br>Ethics Committee, Clinical Hospital Center “Bezanijska Kosa”, Autoput BB, 11070 Belgrade
Slovakia	Eticka komisia, Slovensky ustav srdcovych a cievnych chorob, Pod Krasnou horkou 1, 833 46 Bratislava<br>Eticka komisia, Specializovana nemocnica sv. Svorada ZOBOR, n.o., Klastorska 134, 949 88 NITRA-ZOBOR<br>Ethics Committee of Teaching Hospital Nitra, Spitalska 56, 950 01 Nitra<br>Multicentre Ethics Committee, Teaching Hospital & Policlinic of akad. L. Derer’s, Limbova 5, 833 05 Bratislava
Slovenia	The National Medical Ethics Committee, University Medical Centre Ljubjana, Zaloska 7, 1525 Ljubljana
South Africa	South African Medical Association Research Ethics Committee, Block F Castle Walk, Corporate Park, Nossob Street, Erasmuskloof Ext 3, Pretoria 0153<br>Faculty of Health Sciences Research Ethics Committee, University of Pretoria, Soutpansberg Road, MRC – Building, Room 2-19, Pretoria, Gauteng

Country	Institutional Review Board/Ethics Committee
Spain	Comite Etico Regional de la Comunidad de Madrid, Unidad de Bioetica, P Recoletos, 14-7 planta, 28001 Madrid<br><br>Comite Etico de Investigacion Clinica, Hospital General Universitario de Valencia, Avda. Tres Cruces, s/n, 46014 Valencia<br><br>Comite Etico de Investigacion Clinica, Hospital Ramon y Cajal, Ctra. De Colmenar, Km 9,1, 28034 Madrid<br><br>Comite Etico Investigacion Clinica de Galicia (SERGAS), Division de Farmacia y Productos Sanitarios, Conselleria de Sanidade, c/ San Lazaro, s/n, 15703 Santiago de Compostela<br><br>Comite Etico de Investigacion Clinica, Fundacion Jimeniz Diaz, Avda. Reyes Catolicos, 2, 28040 Madrid<br><br>Comite Etico Investigacion Clinica, Fundacion Hospital Alcorcon, c/Budapest, s/n 28922 Alcorcon (Madrid)<br><br>Comite Etico de Investigacion, Clinica de Aragon, Avda. Gomez Laguna, 25; planta 3, 50009 Zaragoza<br><br>Comite Etico de Investigacion Clinica, Hospital Universitario de Salamanca, Paseo de San Vicente, 182, 37007 Salamanca<br><br>Comite Etico de Investigacion Clinica, Hospital General Universitario Gregorio Maranon, C/ Dr. Esquerdo, 46, 28007 Madrid<br><br>Ethic Committee of Clinical Research Regional de Madrid, C/ Aduana, 29 3º Planta, 28013 Madrid
Thailand	Ethics Committee, Faculty of Medicine Siriraj Hospital, Mahidol University, 2 Prannok Road, Bangkoknoi, Bangkok<br><br>The Khon Kaen University Ethics Committee for Human Research, 123 Mitraphab Road, Dean office 6th Floor, Faculty of Medicine, Khon Kaen University, 123 Mitraphab Road, Muang, Khon Kaen<br><br>The Ethical Review Committee for Research in Human Subjects, Ministry of Public Health, Tiwanon Road, Muang, Nonthaburi<br><br>Research Ethics Committee, Faculty of Medicine, Chiang Mai University, 110 Intavaroros Road, Muang, Chiang Mai<br><br>Institutional Review Board of Bamrasnaradura Infectious Diseases Institute, 126 Tiwanon Road, Nothaburi

Country	Institutional Review Board/Ethics Committee
Ukraine	Ethics Committee of State Pharmacological Centre of Ministry of Health of Ukraine, 7 Grushevsky str., 01021, Kyiv<br>Central Ethics Committee of Health Ministry of Ukraine, 5 Narodnogo Opolchennya str., Kyiv, 03680
USA	Human Studies Subcommittee, Syracuse VA Medical Center, 800 Irving Ave., Syracuse, NY<br>WIRB, 3535 Seventh Avenue SW, Olympia, WA 98502-5010<br>Office of Research Affairs, Rush University Medical Center, 1750 West Congress Parkway, PB II Suite 439, Chicago, IL<br>The Institutional Review Board, Cheryl C. Byers, Director, Wake Forest University Health Sciences, Medical Center Boulevard, Winston-Salem, NC<br>Institutional Review Board, The University of Texas Health Center at Tyler, 11937 US HWY 271, Tyler, TX<br>Colorado Multiple Institutional Review Board (COMIRB), 120101 E.Colfax Avenue Rm #2353, Campus Box F-490, Aurora, CO<br>Duke University Health System IRB, 2400 Pratt Street, 9th Floor, Box 2991, Durham, NC 27705<br>Trident Medical Center, 9330 Medical Plaza Drive, Charleston, SC 29406<br>Guthrie Healthcare System Institutional Review Board, One Guthrie Square, Sayre, PA<br>Biomedical Research Alliance of New York, LLC, Institutional Review Board, 225 Community Drive, Suite 100, Great Neck, NY<br>Phelps County Regional Medical Center – IRB, 1000 West Tenth Street, Rolla, MO<br>Sun Health Institutional Review Board, 10515 W. Santa Fe Drive, Sun City, AZ<br>Joint IRB of St. Rita’s Medical Center and SCCI, 730 W. Market Street, Lima, OH<br>IRB of Lima Memorial Hospital, 1001 Bellefontaine Avenue, Lima, OH<br>University of Florida, Health Center Institutional Review Board, P.O. Box 100173, Gainesville, FL<br>Temple University Institutional Review Board, Temple University Health Sciences Center, 3400 North Broad Street (509-00), Philadelphia, PA

Country	Institutional Review Board/Ethics Committee
	DeKalb Medical Center IRB, 2701 North Decatur Road, Decatur, Georgia
	Office of Research Administration, University of California, Irvine, 300 University Tower, Irvine, CA
	Fox Commercial Institutional Review Board, 326 North Seventh Street, Ste. 218, Springfield, IL
	Our Lady of Resurrection Medical Center Institutional Review Board, 5645 West Addison Street, Chicago, IL
	Durham VAMC, 508 Fulton Street, Durham, NC
	Monmouth Medical Center IRB, Department of Medical Affairs, 300 Second Avenue
	Stanley 223, Long Branch, NJ
	SRMC Institutional Review Board, 101 East Wood Street, Spartanburg, SC
	Research Services, Nebraska-Western Iowa Health Care System, 4101 Woolworth Avenue (111D), Omaha, NE
	Akron General Medical Center, Institutional Review Board, 400 Wabash Avenue, Akron, OH
	Wuesthoff Health Systems, 110 Longwood Avenue, MS #4, Rockledge, FL
	Joint Investigation Review Board, 500 West Broadway, Missoula, MT
	Committee for the Use of Human Subjects in Research, Memorial Hospital of Rhode Island, 111 Brewster Street, Pawtucket, RI
	Institutional Review Board, Edward Hospital & Health Services, 801 South Washington Street, Naperville, IL
	Henry Ford Hospital, Institutional Review Board, 2799 W. Grand Blvd., Detroit, MI
	Health Sciences Institutional Review Board, University at Buffalo, 150 Parker Hall, Buffalo, NY
	Maricopa Integrated Health System, Institutional Review Board, 2601 E. Roosevelt Street, Phoenix, AZ
	Genesys Regional Medical Center Institutional Review Board, One Genesys Parkway, Grand Blanc, MI
	Appalachian Regional Healthcare, Inc., Institutional Review Board, System Center – Hazard, 100 Airport Gardens Road, Hazard, KY

Country	Institutional Review Board/Ethics Committee
	The Methodist Hospital Research Institute, Institutional Review Board, 6565 Fannin Street, STB1-14, Houston, TX<br>University at Buffalo, The State University of New York Health Sciences Institutional Review Board, 150 Parker Hall, Buffalo, NY<br>St. Anthony Hospitals Institutional Review Board, 4231 W.16th Avenue, Denver, CO
